# Analysis of medico-social factors for return to work among patients presenting with haematological malignancy (adamantine): results of a ‘pilot study’

**DOI:** 10.1186/s13104-020-05149-4

**Published:** 2020-07-02

**Authors:** Bénédicte Clin, Natacha Heutte, Mathilde Boulanger, Xavier Troussard, Edouard Cornet, Ghandi Laurent Damaj, Véronique Bouvier, Anne-Valérie Guizard, Guy Launoy, Idlir Licaj

**Affiliations:** 1 INSERM U1086 « ANTICIPE », 14033 Caen Cedex, France; 2 Université de Caen Normandie, Caen, France; 3 Occupational Health Department, Service de Santé au Travail et Pathologie Professionnelle, C.H.U. (University Hospital) Côte de Nacre, CHU Caen, Caen, France; 4 CETAPS EA3832, Université de Rouen, Mont-Saint-Aignan, France; 5 Clinical Research Department, Centre François Baclesse, Caen, France; 6 Service d’Hématologie Clinique, CHU de Caen, Caen, France; 7 Registre des Hémopathies Malignes de Basse-Normandie, Caen, France; 8 Department of Community Medicine, Faculty of Health Sciences, The UiT Arctic University of Norway, Tromsø, Norway

**Keywords:** Cancer, Haematological malignancy, Return to work, Occupational reintegration, Occupational health, Social inequalities

## Abstract

**Objective:**

The aim of this study was to describe return to work determinants in patients with haematological malignancy.

**Results:**

This medico-social pilot study included patients with haematological malignancy in the *département* of Calvados, aged 18 to 55 years, diagnosed between 1st January and 31st December 2010 and alive at 1st January 2015. Patients were identified via consultation of the Lower Normandy haematological malignancy Registry. They completed a specially developed self-questionnaire, in addition to validated questionnaires for anxiety-depression, quality of life and fatigue. Of the patients contacted, 50% accepted to participate. The mean age at diagnosis was 49.8 years, and the majority of patients (79.2%) was professionally active at the time of diagnosis. Only 64.9% of subjects had stopped work due to illness. The psychological impact (demonstrated anxiety) was significantly greater in men (p = 0.01). The majority of subjects returned to work after treatment (80.7%) and among them, the mean duration of absence from work was 16.1 months. Only 52.6% of subjects had informed their occupational physician and 56.7% had benefited from a pre-return visit. The satisfactory response rate obtained is promising for the extension of the present project as a prospective multicentric study.

## Introduction

Cancer is a public health issue affecting more than 380,000 subjects every year in France [1, 2]. In 2018, 45,000 new cases of haematological malignancy were diagnosed in mainland France, of which 25,000 (55%) were males [1, 2]. Heavy treatment and prolonged work stoppages are often required. Even if the global incidence of haematological malignancy is increasing, average patient survival is improving thanks to scientific progress, both in terms of screening and overall cancer care [2].

Factors that may impact the return to work, in particular the duration of sick leave and its social and occupational determinants, as well as the possible specific modalities of such return to work and the associated support offered to patients, are poorly documented [3, 4]. The aim of the present study was to describe the modalities involved in returning to work after treatment in patients with haematological malignancy.

## Main text

### Methods

The study population included professionally active patients (patients with a professional status) with haematological malignancy in Calvados, aged 18 to 55 years inclusive at the date of diagnosis of haematological malignancy, diagnosed between 1st January and 31st December 2010 and alive at 1st January 2015. Inclusion criteria associated: patients who were likely to return to work to the same job and patients who changed jobs. Self-employed subjects (with neither employer nor work colleagues) were excluded from the analysis. The identification of patients with haematological malignancy was conducted via consultation of the Lower Normandy haematological malignancy Registry. Consultation of the RNIPP (National Directory for the Identification of Natural Persons) was conducted by investigators from the cancer registry to ascertain the vital status of included patients.

Patients completed a self-questionnaire, accompanied with an information notice. If the patient did not respond within 8 weeks, a reminder was mailed. An identification list was established, associating each patient with an identification number.

The study was approved by the hospital ethics committee (Ethic Board approval no 15.093). All participants received information on the study and provided written informed consent.

Medical data included: date of diagnosis of haematological malignancy; tumour stage at diagnosis (Ann Arbor classification); initial treatment: type and duration of treatment; possible previous history of cancer(s) and/or concomitant cancer; possible recurrence of a previously treated haematological malignancy; comorbidity factors at time of diagnosis and 3 years after diagnosis.

We developed a specific self-questionnaire (Additional file 1: Questionnaire S1), which was sent together with other validated questionnaires on anxiety-depression (validated French version of the HADS, Hospital Anxiety and Depression Scale [5, 6], quality of life, EORTC, European Organisation for Research and Treatment of Cancer QLQ-C30 questionnaire, in its validated French version [7] and fatigue (Multidimensional Fatigue Inventory, MFI).

The specifically developed questionnaire was developed from previous patient interviews as part of multidisciplinary cancer return-to-work consultations. This allowed us to select relevant items to used in the study questionnaire. This questionnaire included data on: patient sociodemographic characteristics (sex, age); socioprofessional data at the time of diagnosis and at the time of study, together with any potential change in this situation (occupied position, type of work contract, socioprofessional category, working conditions - in particular strenuousness and psychosocial risks, specific arrangements); potential socioprofessional difficulties associated with cancer (sick leave, financial support, disability recognition, etc.).

In order to study the factors associated with return to work as well as those related to employment preservation of patients, we compared 2 groups of subjects from our population. Group 1 consisted of subjects who had not ceased their professional activity and those who had resumed their professional activity after diagnosis; group 2 consisted of subjects who had not resumed their professional activity.

We calculated a ‘Job Evaluation Score Prior to Diagnosis’, which is the combination of the scores for each Job Evaluation Item prior to diagnosis. For each item: 0 points if ‘not at all’ or ‘a little’ in items 1, 2, 6, 8; or if ‘moderately’ or ‘completely’ in items 3, 4, 5, 7. 1 mark if ‘not at all’ or ‘a little’ in items 3, 4, 5, 7; or if ‘moderately’ or ‘completely’ in items 1, 2, 6, 8.

Descriptive statistics were produced using Statistical Analysis System (SAS) software, version 9.3. Exploratory data analysis provided, for qualitative variables, their frequency and their 95% confidence interval and, for quantitative variables, the mean, the standard deviation for the mean, the median and the quartiles. All missing data were systematically reported.

Statistical tests and confidence intervals have been calculated with a bilateral α = 5%.

The Chi^2^ test (or Fisher Exact Test depending on expected study population) was used to compare qualitative values and an ANOVA (analysis of variance) or Student’s *t* test (or Kruskal–Wallis and Wilcoxon test depending on specific cases) was used to compare quantitative variables. Results were considered significant if p < 0.05.

### Results

The general characteristics of the 72 patients who accepted to participate are provided in Table 1. The study population consisted of 45.8% women, mean age was 49.8 (9.5) years and the majority of subjects were active at the time of diagnosis, mostly under permanent contracts.

**Table 1:** Study population characteristics (n = 72)

	n	%	Mean (standard deviation)
Gender			
Men	39	54.2	
Women	33	45.8	
Age (years)			49.8 (9.5)
< 50	30	41.7	
≥ 50	42	45.8	
Diagnosis			
Non-Hodgkin’s lymphoma	19	26.4	
Hodgkin’s disease	11	15.3	
Acute myeloid leukaemia	9	12.5	
Multiple myeloma	8	11.1	
Chronic lymphocytic leukaemia	6	8.3	
MGUS	6	8.3	
Acute lymphoid leukaemia	1	1.4	
Chronic myeloid leukaemia	1	1.4	
Other	10	13.9	
Missing data	1	1.4	
Treatments received			
Chemotherapy	45		62.5
Therapeutic abstinence	18		25.0
Transplantation	12		16.7
Surgery	11		15.3
Radiotherapy	2		2.8
Other	10		13.9

*MGUS* monoclonal gammopathy of undetermined significance

Among the active subjects at diagnosis (N, number = 57 (79.2%)), the majority of subjects (75.4%) were working full-time and on fixed day schedules. The average length of service was 15.7 years.

The majority of subjects (71.9%) rated their professional activity prior to diagnosis as ‘completely satisfactory’, with no significant difference between men and women, or between ages. Forty-two percent of the subjects declared that their professional activity involved a high psychological burden (in terms of time constraints and/or large quantities of work) and only 26.3% of subjects described a high physical burden. In terms of psychological support from co-workers, 43.9% of subjects declared themselves as ‘well supported’. 57.9% of patients considered they had sufficient freedom of movement in their work (responsibilities, satisfactory decision-making margins).

Only 64.9% of subjects reported a sick leave at diagnosis, no significant difference being observed in terms of age or sex. The average duration of sick leave for the entire study population was 19.9 months.

Almost half of patients (47.4%) had not informed their occupational physician and 56.7% had benefited from a pre-return visit, no difference being observed for sex. There was an absence of adapted workstation layout for 90% of patients. The majority of subjects had resumed their professional activity in the same company as the one prior to diagnosis (65.2%). The majority of subjects had taken up the same type of employment contract (86.7%), and in the same job (83.3%). Ninety percent of patients benefited from no adjustment to their working conditions. Arrangements for working conditions included: ‘ergonomic adaptation of your workstation’, ‘arrangement of your workstation schedules’ and ‘other’ (Table 2).

**Table 2:** Occupational situation AFTER diagnosis

	Total	Men	Women	p-value*
Disease-associated sick leave				1.0
Yes: n (%)	37 (64.9)	19 (63.3)	18 (66.7)	
No: (%)	18 (31.6)	9 (30.0)	9 (33.3)	
Missing data	2 (3.5)	2 (6.7)	–	
Duration of sick leave (months): mean	*19.9*	22.0	17.7	0.33
Occupational physician informed				0.10
Yes: n (%)	30 (52.6)	14 (46.7)	16 (59.3)	
No: n (%)	13 (22.8)	10 (33.3)	3 (11.1)	
Missing data	14 (24.6)	6 (20.0)	8 (29.6)	
Pre-return consultation				0.42
Yes: n (%)	21 (56.7)	12 (63.2)	9 (50.0)	
No: n (%)	9 (24.3)	3 (15.8)	6 (33.3)	
Missing data: n (%)	7 (18.9)	4 (21.0)	3 (16.7)	
Return to work: n (%)				1.0
Yes	46 (80.7)	23 (76.7)	23 (85.2)	
No	6 (10.5)	3 (10.0)	3 (11.1)	
Missing data	5 (8.8)	4 (3.3)	1 (3.7)	
Return in the same company				0.66
Yes: n (%)	30 (65.2)	15 (65.2)	15 (65.2)	
No: n (%)	6 (13.0)	4 (17.4)	2 (8.7)	
Missing data n (%)	10 (21.7)	4 (17.4)	6 (26.1)	
Return to the same job				1.0
Yes: n (%)	25 (83.3)	13 (86.7)	12 (80.0)	
No: n (%)	5 (16.7)	2 (13.3)	3 (20.0)	
Return with the same employment contract				1.0
Yes: n (%)	26 (86.7)	13 (86.7)	13 (86.7)	
No: n (%)	1 (3.3)	1 (6.7)	–	
Missing data n (%)	3 (10.0)	1 (6.7)	2 (13.3)	
Adapted working hours				1.0
Yes: n (%)	3 (10.0)	2 (13.3)	1 (6.7)	
No: n (%)	27 (90.0)	13 (86.7)	14 (93.3)	

* Chi square or Fisher test

Of those who reported having a current occupation (Additional file 2: Table S1) 67.7% worked full-time. Return to work conditions were considered sufficiently anticipated for 59.2% of subjects, with no significant difference for age and sex.

The majority of subjects rated the current occupational activity as ‘completely’ satisfactory (62.1%), and 10.3% rated it as ‘not at all’ satisfactory. The analysis shows no significant difference for age and sex. The majority of subjects reported feeling more tired at work than before (‘completely’ for 39.3%, ‘moderately’ for 25%, ‘a little’ for 17.8%, and ‘not at all’ for 17.8%), with no significant difference in age and sex.

The majority of subjects reported feeling that they were working ‘the same as before’ when they discovered their disease (71.4%), and 25.0% reported feeling that they were working ‘less than before’ when they discovered their disease, with no significant difference for age or sex. Of those returning to work, 17.8% reported feeling penalised in their work because of their illness. No significant age and sex differences were observed (Additional file 2: Table S1).

Psychological impact was significantly greater in men (demonstrated anxiety disorder 73.0 versus 41.4, p = 0.001), but no differences were observed for age.

Regarding the depression score, 65.6% of subjects had ‘suspected depressive disorders’ and 28.1% had ‘proven depressive disorders’.

The average score for the overall health and quality of life scale was 65.9/100. No significant differences in these items were found for age and sex. For the assessment of fatigue, the average score was 55.5/100. There was no significant difference for sex, age, overall or sub-scale (Additional file 2: Table S1).

There were no significant differences between Group 1 (subjects who had not ceased their professional activity) and group 2 (subjects who had not resumed their professional activity) for sex, age, length of service, type of employment contract, social protection scheme, working time and working hours (Table 3). The mean duration of absence from work among those who returned to work was 16.1 months (3.1) versus 42.2 months (8.2) for the group 2 (p = 0.002).

**Table 3:** Analysis of obstacles to job retention (n = 52)

	Group 1^b^	Group 2^b^	p-value*
Number of subjects	46	6	
Sex			1.0
Men: n (%)	23 (50)	3 (50)	
Women: n (%)	23 (50)	3 (50)	
Mean age (SD*) (years)	49.8 (9.2)	52.5 (4.4)	0.73
Length of service (years) (SD)	16.5 (12.5)	15.8 (7.4)	0.84
Social security scheme			1.0
General: n (%)	31	4	
Farming: n (%)	3	0	
Civil service: n (%)	6	2	
Self-employed: n (%)	2	0	
Other: n (%)	2	0	
Missing data: n(%)	2	–	
Duration of sick leave (months): mean	16.1	42.2	*0.002*
Type of employment contract			1.0
Permanent: n (%)	30	5	
Fixed term: n (%)	5	0	
Temporary: n (%)	2	0	
Other: n (%)	5	1	
Missing data: n (%)	4	–	
Working basis			0.58
Full-time: n (%)	35	4	
Part-time: n (%)	6	1	
Missing data: n (%)	5	1	
Working hours			0.65
Set day hours: n (%)	27	5	
Set night hours: n (%)	1	0	
Morning/afternoon shifts: n (%)	7	1	
Day/night shifts: n (%)	2	0	
Other: n (%)	3	0	
Missing data: n (%)	6	–	
Professional activity considered SATISFYING			1.0
Not at all: n (%)	3	0	
A little: n (%)	1	0	
Moderately: n (%)	10	1	
Completely: n (%)	32	5	
Professional activity considered FULFILLING			1.0
Not at all: n (%)	2	0	
A little: n (%)	3	0	
Moderately: n (%)	14	2	
Completely: n (%)	27	4	
Sensation of FATIGUE at work			0.08
Not at all: n (%)	8	0	
A little: n (%)	19	1	
Moderately: n (%)	12	2	
Completely: n (%)	7	3	
High PSYCHOLOGICAL burden at work			0.07
Not at all: n (%)	6	0	
A little: n (%)	13	0	
Moderately: n (%)	6	4	
Completely: n (%)	20	2	
Données manquantes: n (%)	1	–	
High PHYSICAL burden at work			0.08
Not at all: n (%)	17	2	
A little: n (%)	16	0	
Moderately: n (%)	3	2	
Completely: n (%)	10	2	
Support from co-workers			0.38
Not at all: n (%)	9	0	
A little: n (%)	11	1	
Moderately: n (%)	6	0	
Completely: n (%)	19	5	
Job Evaluation Score Prior to Diagnosis^a^			0,08
≤ 5: n (%)	17	5	
> 5: n (%)	26	1	
Missing data: n (%)	3	–	
Mean MFI score (standard deviation)	51.8 (16.2)	68.5 (6.50)	*0.004*

* Chi square or Fisher test

^a^Sum of the scores for each work evaluation item before diagnosis. For each item: 0 points if ‘not at all’ or ‘a little’ in items 1, 2, 6, 8; or if ‘moderately’ or ‘completely’ in items 3, 4, 5, 7. 1 mark if ‘not at all’ or ‘a little’ in items 3, 4, 5, 7; or if ‘moderately’ or ‘completely’ in items 1, 2, 6, 8

^b^Group 1: subjects who had not ceased their professional activity and those who had resumed their professional activity after diagnosis

^c^Group 2: subjects who had not resumed their professional activity

The ‘Job Evaluation Score Prior to Diagnosis’ (which is the sum of the scores for each Job Evaluation Item prior to diagnosis), was not significantly different for the 2 groups studied (p = 0.08). In contrast, for the assessment of fatigue, the average score was significantly higher among subjects who did not return to work (p = 0.004) (Table 3).

### Discussion

Unemployment after cancer represents a challenge for both the patient and the society [8] especially when the disease affects a young and previously active population. Studies have demonstrated that the absence of professional activity in these patients may, in particular, lead to impaired quality of life and general state of health, with the risked outcome of social exclusion [9–16]. A meta-analysis based on 36 studies focusing on the relationship between cancer and employment status [17, 18], including 20,366 cancer survivors and 157,603 healthy controls, demonstrated a higher proportion of unemployment among subjects with cancer compared to controls (33.8% vs. 15.2%).

We observed that 79.2% of patients were professionally active at the time of diagnosis and only 64.9% had stopped work due to their illness. The majority of subjects returned to work after treatment (80.7%). A study conducted in Denmark among 1741 patients with haematological malignancy between 2000 and 2007 [19] demonstrated that only 1140 subjects (i.e. 65%) returned to work. Another study, including 130 patients with haematological malignancy and treated by haematopoietic cell transplant, professionally active at the time of diagnosis, demonstrated that, among the 88 surviving subjects 5 years later, 60% had returned to full-time and 32% to part-time work. Return to work was significantly higher in men than women (HR 0.54, 95% CI 0.29–0.99) [20]. In our study, we included 6 cases of MGUS: this disease often had a small impact on general status and professional life. This may explain the higher rate of return to work observed in our study.

Other studies have observed that the longer the sick leave, the more difficult the return to work among patients suffering from cancer, hence highlighting the importance of early medico-social intervention [3, 4]. In our study, the mean duration of the absence from work among subjects who returned to work was 16.1 months vs 42.2 months for the group which had not returned to work (p = 0.002). This encourages patients to quickly seek employment adaptations, and to consider well in advance the possible layout of their workstation, in order to anticipate recovery and avoid occupational exclusion.

The role of the occupational physician in the return to work for cancer patients has been previously demonstrated and a significantly higher level of return to work was observed in patients who benefited from arrangements in their working conditions [21, 22]. Unfortunately, according to another study, only one in 4 patients benefited from a consultation with the occupational physician immediately prior to return to work and only 50% benefited from special work arrangements [22]. Similarly, we found that almost half of patients had not informed their occupational physician about their disease and that 56.7% had benefited from a pre-return consultation. As a result, 90.0% of subjects did not benefit from any adjustment to their working conditions.

## Limitations

One of the limitations of our pilot study is its low statistical power, which has not allowed us to analyse other determinants of job retention such as occupational category, education level and income level. Some factors were close to significant (psychological burden and physical burden between subjects who had returned to work or not, in particular). The results of the present pilot study will allow us to make proper statistical power estimation about the size of each working group.

The originality of our study was to exhaustively recruit all subjects with haematologic malignancies from a well-known regional cancer registry. This ensures the completeness and reliability of diagnosis.

This study yielded promising results which led to its extension to include other medical centres. The results of this study should enable the widespread dissemination of information and the development of tools to identify factors of social vulnerability for professionals and associations involved in accompanying patients presenting with haematological malignancy.

## Supplementary information

**Additional file 1.** Study self-questionnaire.

**Additional file 2: Table S1.** CURRENT occupational situation (n=46).

## Data Availability

The datasets used and/or analysed during the current study are available from the corresponding author on reasonable request.

## Abbreviations

ANOVA: Analysis of variance
EORTC: European Organisation for Research and Treatment of Cancer
HADS: Hospital Anxiety and Depression Scale
MFI: Multidimensional Fatigue Inventory
N: Number
RNIPP: National Directory for the Identification of Natural Persons
SAS: Statistical Analysis System
SD: Standard deviation
