# Supplementary material for: Analysis of medico-social factors for return to work among patients presenting with haematological malignancy (adamantine): results of a ‘pilot study’
Source: BMC Res Notes. 2020 Jul 2;13:313. doi: 10.1186/s13104-020-05149-4 (PMC7331231; doi:10.1186/s13104-020-05149-4)
Supplement: Supplementary file 1 — Additional file 1. Study self-questionnaire. [file 13104_2020_5149_MOESM1_ESM.docx]

**Additional file : Study self-questionnaire**


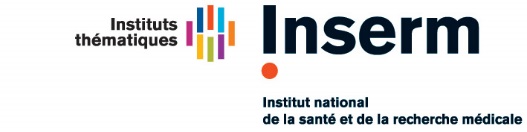

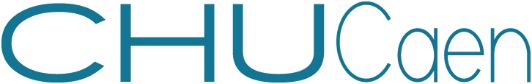


Observational research

**Anonymous study number :**

**Year of birth |__|__|__|__|**

**Sexe 🞏 Men 🞏 Women**

ANALYSIS OF MEDICO-SOCIAL FACTORS FOR RETURN TO WORK AMONG PATIENTS PRESENTING WITH HAEMATOLOGICAL MALIGNANCY: ADAMENTINE pilote study

**①** Were you working at the time of diagnosis ?

🞏 Yes (go to section ➁) 🞏 No

If not, were you:

🞏 unemployed

🞏 on disability

🞏 in training, specify : …….……..

🞏 at home

🞏 other, specify: ………….…….…..

Specify in section ➂

Professional situation **before** diagnosis:

**➁**

- What was your profession ? ………………………………………
- What was the activity sector of your company ?………………………………….
- The company was ?

🞏 ≤10 employees 🞏 between 10 & 250 employees

🞏 > 250 employees

- Company sector  ?

🞏 private 🞏 public 🞏 private involved in public sector

- Social security system :

🞏 general security system

🞏 agricultural system

🞏 public system

🞏 self-employed, non-agricultural

🞏 other system, specify:……………………………

- What was your seniority ? |__|__| 🞏 months 🞏 years
- Family situation ?

🞏 childless 🞏 with child, if yes number : ……….

🞏 in relationship 🞏 single

**➁ continuation**

- Type of contract : 🞏 Limited-term contract 🞏 temporary work 🞏 permanent contract 🞏 internship 🞏 student

🞏 other, specify ………………

- Working time ?
- 🞏 full-time 🞏 part-time (if yes, specify : |__|__| %)
- Were you working from home ?
- 🞏 yes 🞏 no
- What were your working hours ?

🞏 daytime fixed working hours 🞏 discontinued morning and afternoon

🞏 fixed at night 🞏 discontinued day/night 🞏 other

- Were you on sick leave 🞏 yes 🞏 no

If yes since when ? …………………..

If yes was it related to your hematological malignancy ? 🞏 yes 🞏 no

- Were you happy with your profesionnal activiy?

🞏 not really 🞏 a little 🞏 moderatly 🞏 absolutely

- Did you feel fulfilled at work?
- 🞏 not really 🞏 a little 🞏 moderatly 🞏 absolutely
- Did you feel tired at work?
- 🞏 not really 🞏 a little 🞏 moderatly 🞏 absolutely
- Did your work have a high psychological demand (time constraint and or signifivant amount of work) ?
- 🞏 not really 🞏 a little 🞏 moderatly 🞏 absolutely
- Did your work involve a heavy physical load
- 🞏 not really 🞏 a little 🞏 moderatly 🞏 absolutely
- Did you receive satisfactorysupport from your work colleagues ?
- 🞏 not really 🞏 a little 🞏 moderatly 🞏 absolutely
- Did you find the working atmosphere as good ?
- 🞏 not really 🞏 a little 🞏 moderatly 🞏 absolutely
- Did you find the relations with your colleagues satisfactory ?
- 🞏 not really 🞏 a little 🞏 moderatly 🞏 absolutely

**➁ Etiez-vous :**

**🞏 au chômage**

**🞏 en invalidité**

**🞏 en formation, précisez : ……………………………………..**

**🞏 au foyer**

**🞏 autre, précisez : ………………………………………………..**

**➁ suite**

- Did you need help from your co-workers to complete certain tasks ?
- 🞏 not really 🞏 a little 🞏 moderatly 🞏 absolutely
- Did the manager have flexibility in terms of employee managment ?
- 🞏 not really 🞏 a little 🞏 moderatly 🞏 absolutely
- Was your company being restructured

🞏 yes 🞏 no

- There were a union presence in your work ?

🞏 yes 🞏 no

- Did you have certain autonomy in your work (responsibilities, sastisfactory decision-making margins)?
- 🞏 not really 🞏 a little 🞏 moderatly 🞏 absolutely

**➂**

Have you been off work (or on sick leave) at the discovery of your illness

🞏 yes (go to section ➃) 🞏 no (go to section ➄)

**PROFESSIONAL SITUATION AFTER DIAGNOSIS :**

**➃**

- Are you still on seick leave now?

🞏 yes 🞏 no

- How long were you on sick leave ?

|__|__| 🞏 days 🞏 months

- Did you inform your familly doctor about the diagnosis ?
- 🞏 yes 🞏 no

If not why ?

🞏 No need or interest

🞏 fear of consequences

🞏 unaware of the possibility

🞏 other, specify ………………………..

**➄**

**If your resumed your work after the seack leave (or continued working withour a sick leave):**

- Have you met your occupational doctor on a pre-recovery visit?

🞏 yes 🞏 no

If yes, how many time ? |__|__|

On the advice of which person ?…………………..……………………………

- Have you taken over in the same company ?

🞏 yes (go to section ➅) 🞏 no

If not : why ?

🞏 end of contract

🞏 resignation, specify for what reason(s) :

🞏 change of priorities

🞏 health issues

🞏 failure to obtain requested accommodation

🞏 transport difficulties

🞏 relational difficulties in the workplace

🞏 other job

🞏 other specify : …………………………………….

🞏 dismissal, why :

🞏 economic reasons

🞏 medical incapacity

🞏 other specify : ………………..………….

🞏 bankruptcy

🞏 autre, précisez : ……………….……………….

**➅**

- **If you took over in the same company, did you took over at the same position** ?

🞏 yes 🞏 no

If not : what position ? …………………………………….

- Did you resume with the same type of employment contract

🞏 yes 🞏 no

If no : with what type pf employment contract ?

🞏 Limited-term contract 🞏 temporary work 🞏 permanent contract 🞏 other, specify……………..

- What were the terms of your recovery

🞏 full time 🞏 part-time, including therapeutic part-time (specify : |__|__|%)

- What were the working hours

🞏 fixed during the day 🞏 posted morning afternoon

🞏 fixed during night 🞏 posted day/night 🞏 other

- Have you benefited from any accommodation in your working conditions

🞏 yes 🞏 no

If yes , specify ?

🞏 medical restriction (specify why : ………………………………)

🞏 ergonomic layout of your workstation

🞏 sheduling of your work station

🞏 professional reclassification

🞏 other (specify ………………………..)

- Have you noticed other impacts of your illness on your work ?

🞏 yes 🞏 no

If yes, specify which ?

🞏 drop in income

🞏 dismissed by colleagues and/or the hierarchy

🞏 loss of career advancement

🞏 other (specify ……………….…………..)

- After resuming your professional activity, have you had one or more stoppage(s) related to your illness

🞏 yes 🞏 no

If yes, how many day or months in total |__|__| 🞏 days 🞏 months

**If you have not returned to work :**

- Would you like to return to work
- 🞏 not really 🞏 a little 🞏 moderatly 🞏 absolutely
- Why have you not returned to work ?

🞏 your feel too tired

🞏 your are still being treated for your ilness

🞏 you are in pain or with limited physical capacities,

If yes, specify which one ?.............................................................

🞏 other reason(s) (financial, family,…), specify..............................................

**In bothe cases returned or not to work:**

- Have one or more of these steps been taken

🞏 Recognition as a disabled worker

🞏 recognition as invalid : ………..

🞏 Long term leave or long term seack leave

🞏 dismissal for medical incapacity

🞏 eraly retirement or retirement

🞏 request for allowance for disabled adults

🞏 asked support from existing options at your work

🞏 support by the out of work special service (work support for disabled)

🞏 support by social service

🞏 help for retraining

🞏 other, specify ……………………

**CURRENT PROFESSIONAL SITUATION :**

**Currently, if you have resumed your professional activity :**

Do you work full time ?

🞏 yes 🞏 no

If not , why ?

🞏 personal choice

If yes :

🞏 for a reason related to your illness

- 🞏 for family reasons

🞏 part-time therapeutic ongoing since |__|__|/|__|__|/|__|__|.

🞏 invalidity

- Are you staistied that you have returned to work ?
- 🞏 not really 🞏 a little 🞏 moderatly 🞏 absolutely
- Do you think your return to work was anticipated
- 🞏 not really 🞏 a little 🞏 moderatly 🞏 absolutely
- Do you find your professinal activity satisfactory ?
- 🞏 not really 🞏 a little 🞏 moderatly 🞏 absolutely
- Do you feel fulfilled at work ?
- 🞏 not really 🞏 a little 🞏 moderatly 🞏 absolutely
- At work do you feel more tired than before ?
- 🞏 not really 🞏 a little 🞏 moderatly 🞏 absolutely
- Do you feel supported from your work colleagues ?
- 🞏 not really 🞏 a little 🞏 moderatly 🞏 absolutely
- Do you need help from your co-workers to complete certain tasks ?
- 🞏 not really 🞏 a little 🞏 moderatly 🞏 absolutely
- DO you feel that your are working

🞏 worse than before 🞏 the same as before 🞏 better than before

**Currently, if you have resumed your professional activity :**

- Do you feel that you have been penalized in your job because of your illness

🞏 no 🞏 yes, a bit 🞏 yes, a lot

If yes, specify how :

🞏 demotion

🞏 promotion refused

🞏 loss of responsability

🞏 unwanted mutation

🞏 loss of benefit

🞏 Unwanted reassignment of tasks

🞏 rescheduled working hours

🞏 other precise : ………………………………………………………….

- Some hospitals offer multidisiplinary return-to-work assistance, consultataions combining a specialized doctor (occupational pathology), a psychologist and a social worker. Have you ever benefited from such a consultation ?

🞏 yes 🞏 no

- Would you like to benefit (again or for the first time) from such a consultation?

🞏 yes 🞏 no
