# Supplementary material for: Analysis of medico-social factors for return to work among patients presenting with haematological malignancy (adamantine): results of a ‘pilot study’
Source: BMC Res Notes. 2020 Jul 2;13:313. doi: 10.1186/s13104-020-05149-4 (PMC7331231; doi:10.1186/s13104-020-05149-4)
Supplement: Supplementary file 2 — Additional file 2: Table S1. CURRENT occupational situation (n=46). [file 13104_2020_5149_MOESM2_ESM.docx]

**Additional file: table S1**

**Table S1 – CURRENT occupational situation (n=46)**

|  | **Total** | | **Men** | | **Women** | | | **p- value*** |  |
| --- | --- | --- | --- | --- | --- | --- | --- | --- | --- |
| **SATISFIED with return to work**   - Not at all: n (%) - A little: n (%) - Moderately: n (%) - Completely: n (%) - Missing data n (%) | -  -  4 (8.7)  31 (67.4)  11 (23.9) | | -  -  1 (4.3)  15 (65.2)  7 (30.4) | | -  -  3 (13.0)  16 (69.6)  4 (17.4) | | | 0.61 |  |
| **Return sufficiently ANTICIPATED**   - Not at all: n (%) - A little: n (%) - Moderately: n (%) - Completely: n (%) - Missing data n (%) | 3 (6.5)  -  10 (21.7)  20 (43.5)  13 (28.3) | | -  -  5 (21.7)  9 (39.1)  9 (39.1) | | 3 (13.0)  -  5 (21.7)  11 (47.8)  4 (17.4) | | | 0.37 |  |
| **MORE TIRED than before**   - Not at all: n (%) - A little: n (%) - Moderately: n (%) - Completely: n (%) - Missing data n (%) | 6 (13.0)  8 (17.4)  7 (15.2)  14 (30.4)  11 (23.9) | | 4 (17.4)  4 (17.4)  1 (4.3)  7 (30.4)  7 (30.4) | | 2 (8.7)  4 (17.4)  6 (26.1)  7 (30.4)  4 (17.4) | | | 0.27 |  |
| **Support from co-workers**   - Not at all: n (%) - A little: n (%) - Moderately: n (%) - Completely: n (%) - Missing data n (%) | 7 (15.2)  5 (10.9)  8 (17.4)  14 (30.4)  12 (26.1) | | 4 (17.4)  4 (17.4)  3 (13.0)  5 (21.7)  7 (30.4) | | 3 (13.0)  1 (4.3)  5 (21.7)  9 (39.1)  5 (21.7) | | | 0.36 |  |
| **Impression of being PENALISED by illness**   - No: n (%) - Yes, a little: n (%) - Yes, a lot^‡^ : n (%) - Missing data n (%) | 29 (63.0)  2 (4.3)  5 (10.9)  10 (21.7) | | 14 60.9)  1 (4.3)  4 (17.4)  4 (17.4) | | 15 (65.2)  1 (4.3)  1 (4.3)  6 (26.1) | | | 0.66 |  |
| **HADS anxiety depression scale**  **Anxiety score**   - No anxiety: n (%) - Suspected anxiety: n (%) - Confirmed anxiety: n (%)   **Depression score**   - No depression: n (%) - Suspected depression: n (%) - Confirmed depression: n (%) | | 9 (13.6)  18 (27.3)  39 (59.1)  10 (14.5)  46 (66.7)  13 (18.8) | | 5 (13.5)  5 (13.5)  27 (73.0)  8 (21.6)  25 (67.6)  4 (10.8) | | 4 (13.8)  13 (44.8)  12 (41.4)  2 (6.2)  21 (65.6)  9 (28.1) | **0.01**  0.07 | | |
| **Quality of Life (QLQ-C30) (mean/100)**   - Functional capacity - Capacity to accomplish any occupation or leisure activity - Emotional state - Cognitive capacity - Capacity to maintain social relationships   Overall result | | 81.9  81.2  71.6  80.0  76.5  **67.5** | | 84.3  82.0  76.5  82.0  7408  **68.8** | | 79.2  80.2  65.6  77.6  78.6  **65.9** | 0.59 | | |
| **MFI fatigue scale (moyenne/100)** | | 54.1 | | 53.1 | | 55.5 |  | | |

* Chi-square or Fisher test

** Standard Deviation

‡ type of penalisation : demotion for n=1 subjet, loss of benefits for n=1, other for n=4
